# Resilience as a dynamic process among military recruits exposed to basic combat training stressors

**DOI:** 10.1017/S0954579426101199

**Published:** 2026-02-18

**Authors:** Mollie A. McDonald, Craig A. Marquardt, Siamak Noorbaloochi, Emily Hagel Campbell, Ann S. Masten, Melissa A. Polusny

**Affiliations:** 1 VA Salisbury Healthcare System, USA; 2 Minneapolis VA Health Care System, USA; 3 Psychiatry and Behavioral Sciences, University of Minnesota, USA; 4 VA Center for Care Delivery and Outcomes Research, USA; 5 Institute of Child Development, University of Minnesota Twin Cities, USA

**Keywords:** adversity, military, cognitive ability, internalizing, resilience, self-regulation

## Abstract

New military recruits, typically emerging adults, must rapidly adapt to the stressors of basic combat training (BCT) – a developmentally significant and intentionally stressful experience. Drawing on a developmental psychopathology framework of risk and resilience, we prospectively examined predictors of psychological adaptation in a longitudinal sample of recruits (age mean = 19.0, *SD* = 3.0) assessed before and after BCT (59.7% of those eligible for follow-up; *N* = 657). Pre-registered hierarchical linear regressions tested direct and moderating effects of individual difference variables previously linked to risk and resilience. Higher levels of prior adversity, worse self-regulatory difficulties, and (unexpectedly) higher general cognitive ability at baseline were associated with worsening post-BCT internalizing distress, after accounting for baseline symptoms. Gender, baseline social support, and baseline Multidimensional Personality Questionnaire (MPQ) scales were not associated with longitudinal changes in internalizing distress, and no moderation effects were found. Our findings suggest that bolstering emotion regulation skills, especially among those with prior adversity, may be important for preventing the emergence of psychopathology and promoting more successful adaptation to military roles. The unexpected association between cognitive ability and distress may reflect context sensitivity, suggesting that the demands of BCT may alter the typical adaptive function of cognitive strengths.

For new recruits in military settings, enlistment marks the start of an intense developmental period of role transitions, acculturation, and personal challenges. This is due in part to the rapid onset of stressors distinctive to military life. The 10-week United States (US) Army Basic Combat Training (BCT) program is a significant and demanding period of this adjustment. Recruits often face challenges due to the program’s intense physical demands, rigid discipline, and carefully managed exposure to stress-inducing situations (Williams et al., 2016). Although many recruits successfully adapt to these demands, nearly one in five does not complete BCT, preventing them from entering full military service (United States Government Accountability Office, 2015). To evaluate potential resilience factors supporting positive responses to BCT, we evaluated longitudinal changes in internalizing symptoms among military recruits following their initial entry into the National Guard. In particular, we assessed the impact of recently experienced BCT stressors, and explored the ways in which individual differences may shape mental health outcomes.

We applied principles from developmental psychopathology to investigate resilience among this military population ( Luthar et al., 2000; Masten et al., 2021; Masten & Cicchetti, 2016). Though originally designed for studies of children, developmental models of psychopathology and resilience have been expanded to study adjustment across the lifespan (Bonanno et al., 2015; Burt & Paysnick, 2012a). Explorations of outcomes within new contexts, such as military service, may reveal nuances in how resilience processes unfold over time. For instance, certain aspects of resilience such as the quality of parenting are especially significant during childhood, while other factors – like support from people outside of the home – tend to play a larger role as people grow older, adding to influences that persist from early life (Seok & Doom, 2022). Resilience in military roles may jointly stem from broadly applicable resilience factors helpful under many circumstances (e.g., cognitive ability) as well as new processes more specific to military life (e.g., military unit support). Examination of BCT offers a unique addition to resilience science by assessing responses to a well-defined period of intense stressors in naturalistic contexts as opposed to uncontrolled stressors or experimentally administered stressors with limited generalizability (Polusny et al., 2021). Thus, we aimed to test existing models of resilience derived from non-military contexts within a large sample of military recruits exposed to BCT.

Resilience comes about from a dynamic capacity to adapt successfully to challenges, which manifests through processes that unfold over time. This is possible when various adaptive systems within (e.g., self-regulatory abilities) and around (e.g., social support) an individual are activated (Luthar et al., 2000; Ungar & Theron, 2020; Yates et al., 2015). These systems exert their effects by facilitating a person’s capacity to continue functioning well in their current circumstances or to recover despite adversity. Therefore, accurate measurements of longitudinal changes in adaptive functioning as well as separate assessments of potential resilience and risk factors are the central ingredients for research on resilience processes (Masten et al., 2021; Polusny et al., 2023). In contrast, cross-sectional examinations of stressor effects cannot reveal resilience-related outcomes due to a lack of information about relative changes in adjustment over time (Bonanno et al., 2015; Polusny et al., 2017). Importantly, most research studies of military stress to date have relied on cross-sectional designs or have operationalized resilience as a cross-sectionally measured unidimensional trait (van der Meulen et al., 2020), thereby limiting our understanding of resilience as a dynamic process. The current study builds on existing military research by implementing a pre–post design and careful assessments of several individual difference domains thought to confer the capacity for resilience.

Models of resilience make a distinction between promotive/risk and protective/vulnerability processes, reflecting main versus moderating effects, respectively (see Figure 1). This is in alignment with the distinction between various stressor-independent and stressor-dependent effects often discussed within the resilience literature (Hankin & Abela, 2005; Masten et al., 2021) and the rich conceptual theorizing of Suniya Luthar and colleagues (Luthar et al., 2000), which this special issue honors. Deleterious stressor-independent effects happen when attributes and experiences with negative consequences on adjustment (e.g., preexisting risk factors) combine with the stressors to increase the likelihood for future psychopathology (i.e., summed main effects across risk factors and stressors). In contrast, stressor-dependent effects can occur when preexisting vulnerability factors facilitate the later emergence of psychopathology by exacerbating stressor effects (i.e., moderation). Resilience models also allow for promotive factors, which have positive main effects on adjustment in ways that offsets some of the negative effects of risk factors and stressors. Finally, protective factors can be observed, which moderate and buffer against the negative effects of stressors (Garmezy et al., 1984; Masten et al., 2021).

**Figure 1.**
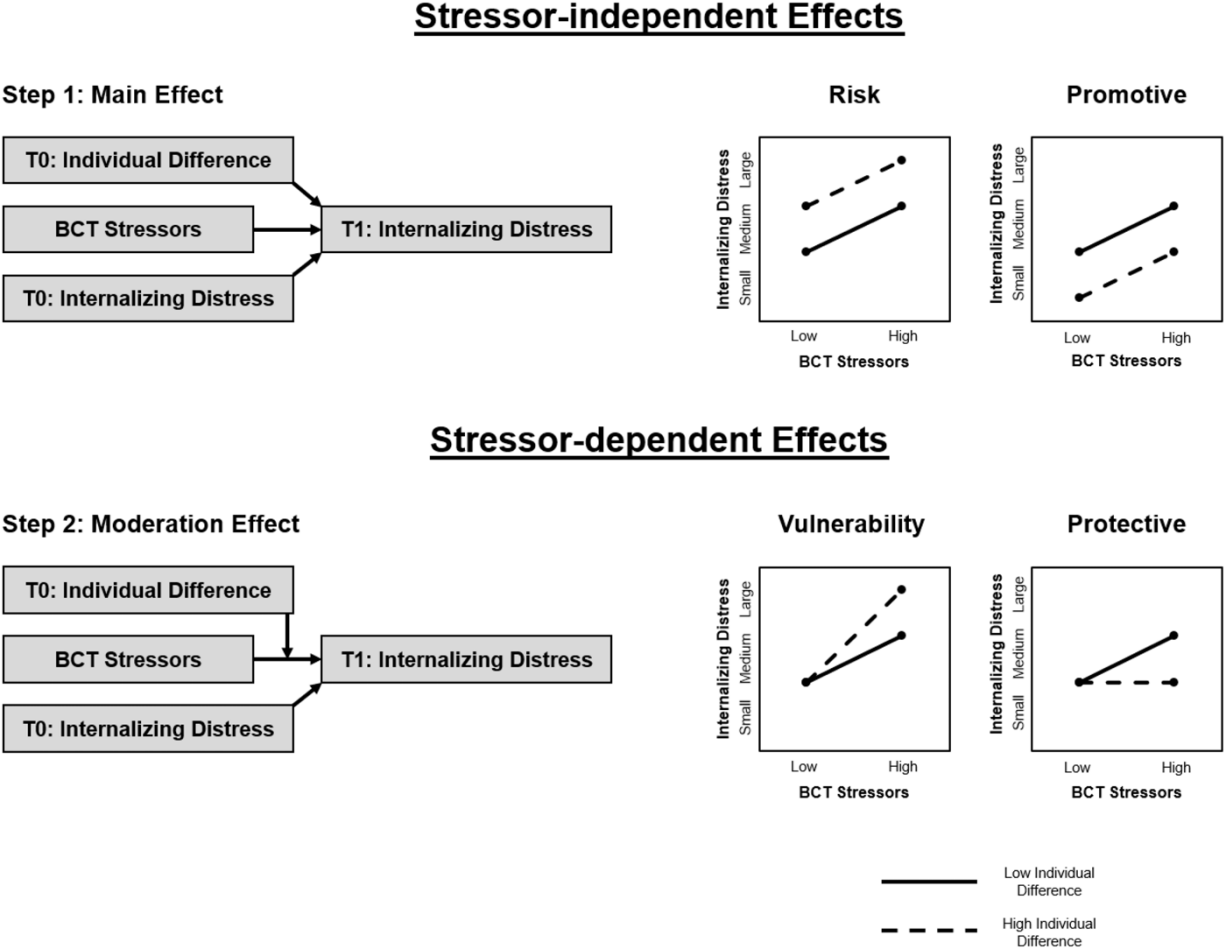
Developmental psychopathology framework for the study of resilience in military recruits exposed to BCT stressors. *Note*. BCT = basic combat training; T0 = time 0 (pre-BCT baseline); T1 = Time 1 (post-BCT follow-up). Conceptual design of stressor-independent and stressor-dependent longitudinal effects of BCT stressors modeled using a developmental psychopathology statistical and theoretical framework.

In the broader resilience literature, numerous predictors have been identified as important for shaping outcomes throughout development including self-control, problem-solving skills, and supportive relationships (Cicchetti & Garmezy, 1993; Masten et al., 2021; Masten & Narayan, 2012). Among adults in civilian settings, adverse childhood experiences are known to predict worse functional outcomes as well such as greater emotional distress and internalizing symptoms (Reich et al., 2012). Young women may be particularly vulnerable following stressors given the known gender differences in internalizing psychopathology that emerge during this age window (Hankin et al., 1998; Meadows et al., 2006). Several attributes have also been consistently identified as resilience resources contributing to better outcomes, even among young adults exposed to prior adversity as children: coping skills, cognitive skills, supportive relationships, and early adaptive behavioral characteristics (i.e., an “easy” temperament at birth; (Burt & Paysnick, 2012b; Fritz et al., 2018; Masten, 2001; Sapienza & Masten, 2011; Singh & Wendt, 2024). Furthermore, personality traits such as negative emotionality (neuroticism), constraint (conscientiousness), and positive emotionality (PEM) (extraversion) often predict an assortment of adjustment outcomes in civilian populations (Masten et al., 1999; Roberts et al., 2007; Shiner & Masten, 2012). Among current and former military service members, there may be similar factors shaping the likelihood of developing posttraumatic stress and internalizing symptoms, including neuroticism, lack of social support, maladaptive coping habits, and female gender (Pavlacic et al., 2022). Yet, the relevance of these assorted factors for explaining longitudinal risk and resilience among young military recruits following BCT is largely unknown.

Although many individual differences appear to exhibit main effects on outcomes in resilience models, fewer show consistent moderation effects (Shiner & Masten, 2012). Additionally, given the previously mentioned methodological limitations within the existing military resilience research (e.g., cross-sectional designs; van der Meulen et al., 2020), it is even less clear which resilience factors lead to moderation effects in military populations. Moderation within longitudinal designs suggests that there are individual differences shaping the impact of the stressors themselves rather than just offsetting stressor effects. Therefore, searching for moderating effects is a key step toward designing public health efforts to enhance protective resilience processes and reduce vulnerability to environmental challenges, which could lead to interventions testable through resilience-informed randomized controlled trials (Chmitorz et al., 2018; Luthar & Brown, 2007; Yates et al., 2015). Military service members may have a heightened need for this kind of resilience research given their elevated reporting of many life stressors (Blosnich et al., 2014).

## Present study

The goal of this pre-registered study (https://osf.io/csf4r) was to examine resilience as a dynamic process in relation to the development of internalizing psychopathology among emerging adults undergoing BCT. The study addresses three key objectives: (1) test the longitudinal associations between BCT stressor exposures and relative worsening of internalizing symptoms, (2) evaluate potential risk and vulnerability factors such as negative emotionality, self-regulation difficulties, gender, and lifetime stressor/adversity loading, and (3) examine potential promotive and protective factors, including cognitive ability, PEM, constraint, and social support. Using established hierarchical regression modeling techniques that distinguish between main and moderation effects (e.g., Garmezy et al., 1984; Marquardt et al., 2023), we assessed how individual differences shape responses to BCT stressors above-and-beyond the effects of the stressors themselves. The structured and time-bound nature of BCT provided a naturalistic, experimental-like framework to systematically study resilience as a dynamic process among military recruits. Findings from this study may enhance our understanding of resilience mechanisms and inform future efforts aimed at mitigating internalizing psychopathology in high-stress environments.

## Methods

### Participants and procedure

Data were drawn from the Advancing Research on Mechanisms of Resilience (ARMOR) study, a prospective 5-wave longitudinal study of military recruits over the first two years of military service. The initial baseline assessment occurred before recruits shipped for BCT (T0; years 2019–2021); follow-up began two weeks after each participant returned from BCT (T1; years 2019–2023) and continued 6, 12, and 18 months later (see Polusny et al., 2023, for detailed description of study design and protocol). The first two measurement timepoints (i.e., T0, T1) were the specific focus of this report.

At the T0 baseline, new recruits aged 17 years or older attending drill training at National Guard armories were invited in person to participate. Soldiers were excluded if they had previously been exposed to BCT or a parent opted them out of the study. After informed consent and assent, recruits could choose to complete computerized self-administered measures of mental health symptoms and a range of risk and resilience factors. Follow-up T1 post-BCT measures were administered via Qualtrics; paper-and-pencil versions were sent by mail to non-responders.

**Figure 2.**
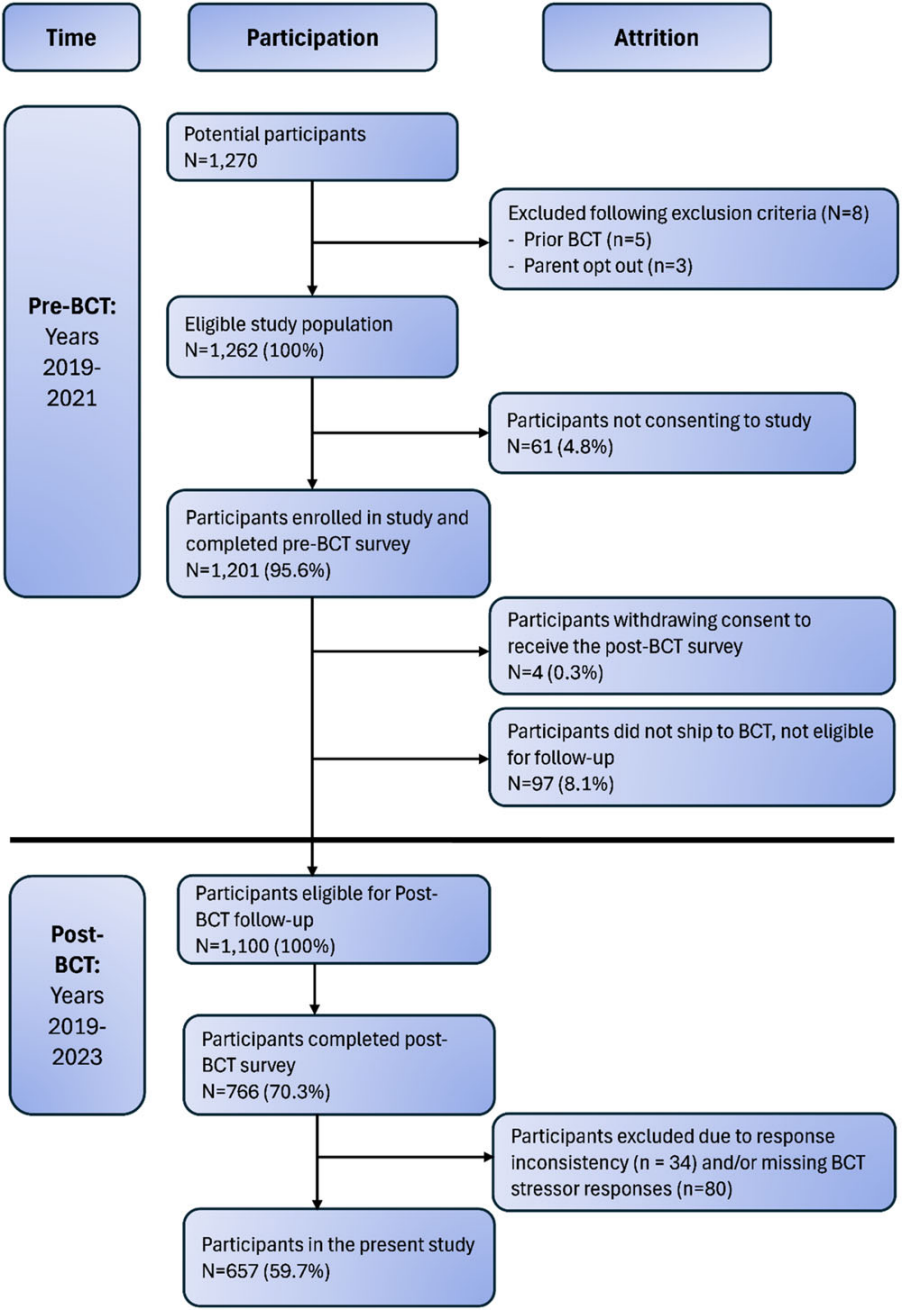
Participant flow chart. *Note*. BCT = basic combat training.

A total of 1,270 recruits were approached and invited to participate in the study (8 were ineligible following exclusion criteria). Of the 1,262 eligible recruits at T0, 1,201 (95.6%) agreed to participate (age *M* = 19.0, *SD* = 3.0). Four participants withdrew their consent to be contacted for follow-up, and 97 participants failed to ship to BCT and thus were ineligible for follow-up. Of the eligible T0 participants who shipped to BCT (*n* = 1,100), 766 (70.3% of eligible) completed the T1 survey. Participants were excluded from analyses if they demonstrated response inconsistency on the Multidimensional Personality Questionnaire-155/Brief Form (MPQ-155/BF), and/or did not provide sufficient responses to questionnaires assessing internalizing psychopathology and BCT stressors (i.e., responded to less than 80% of items). This resulted in an analysis sample of 657 recruits (59.7% of eligible; see Figure 2) for the present study.

Attrition analyses between the study sample (*n* = 657) and eligible participants who did not provide sufficient study data (*n* = 443) revealed few baseline differences at T0. The study analysis sample produced lower T0 MPQ PEM raw scores (*M* = 71.0, *SD* = 14.9) than recruits with insufficient T1 data (*M* = 72.8, *SD* = 14.4), *t* (1079) = 1.97, *p* = .049. Also, the study analysis sample had higher estimates of general cognitive ability (*M* = .103, *SD* = .846) than recruits with insufficient T1 data (*M* = −.130, *SD* = .827). However, the effect sizes were small in magnitude (Cohen’s *d* = .12 and −.28, respectively). There were no statistically significant differences between the two groups based on other study measures of potential resilience capacity or internalizing psychopathology at T0 (*ps* > .06; Tables S1 and S2).

All participants provided informed consent or assent. For participants under age 18, IRB approved a waiver of parental informed consent because participants were military personnel. The study was approved by the institutional review boards of the University of Minnesota (STUDY00004470) and Minneapolis VA Health Care System (VAM-18-00334) as well as the relevant military command.

### Measures

#### Internalizing distress

Consistent with a dimensional, hierarchical conceptualization of psychopathology (Watson et al., 2022), we modeled T0 and T1 internalizing distress as a higher order latent construct (see modeling details in Supplemental Materials) by estimating the common variance across the following four indicator measures: Patient-Reported Outcomes Measurement Information System (PROMIS) Depression-4, PROMIS Anxiety-4, PROMIS Anger-5 scales (Cella et al., 2010; Pilkonis et al., 2011), and the Primary Care PTSD Screen for DSM-5 (PC-PTSD-5; Prins et al 2016). Internal consistency of the three PROMIS Depression-4, Anxiety-4, and Anger-5 scales was good-to-excellent at T0 and T1 (Cronbach’s *α* = .87 − .94). For the PC-PTSD-5, internal consistency was good (Cronbach’s *α* T0 = .81, T1 = .80).

#### Stressor exposure

The Basic Training Stressor Scale (BTSS), developed for this project and validated in prior published work (Polusny et al., 2021), assessed recall at T1 of self-reported challenges commonly experienced during BCT. For this study, we calculated a BTSS total score by summing frequency and impact scores.

#### Personality

At baseline (T0), the MPQ-155/BF (Patrick et al., 2002) was used to measure three broad dimensions of normal-range personality (Positive Emotionality [PEM], Negative Emotionality [NEM], Constraint [CON]) together with Absorption. PEM is a measure of tendencies toward positive emotions (e.g., wellbeing) and active engagement in social and work pursuits; NEM measures the tendency toward negative emotions, exaggerated stress reactions, and adversarial interpersonal relations; and CON reflects the tendency toward planful, cautious, and conventional attitudes and behaviors. We used established weighting equations to generate the NEM, PEM, and CON estimates from primary trait scales. Absorption is a stand-alone 12-item primary trait scale with similarities to openness to experience without the confound of political liberalism. Valid profiles were determined using embedded response inconsistency indices with the cut-score recommendations from Patrick and colleagues (2002). We excluded invalid profiles (*n* = 20) from analyses.

#### Self-regulation

The 16-item version of the Difficulties in Emotion Regulation Scale (DERS-16; Bjureberg et al., 2016) was used at T0 to assess difficulties with awareness and understanding of emotions, acceptance of emotions, behavior in accordance with goals despite negative emotions, and using effective emotion regulation strategies. Higher scores represent greater difficulties in emotion regulation (Cronbach’s *α* = .92).

#### Social support

Social support at T0 was modeled using a latent estimate (see Supplemental Materials for modeling details) across four indicators: PROMIS Instrumental Support-4 (Cronbach’s *α* = .90), PROMIS Informational Support-4 (Cronbach’s *α* = .93), PROMIS Emotional Support-4 (Cronbach’s *α* = .94), and the Unit Social Support scale from the Deployment Risk and Resilience Inventory-2 (DRRI-2; Cronbach’s *α* = .94; Vogt et al., 2013). Together, these scales reflected perceptions of practical support, availability of helpful information from others, emotional dependability of relationships, and military unit cohesion. By using a latent model, we were interested in quantifying the general perceptions of social support common across these various domains for each participant. For all scales, higher scores represented greater social support at baseline before BCT.

#### General cognitive ability

General cognitive ability was modeled using a latent estimate (see Supplemental Materials for modeling details) of cognitive ability at baseline. We used the Armed Forces Qualification Test (AFQT) scores (Plag & Goffman, 1967) as one of the indicators. This paper-and-pencil test of general cognitive ability is taken by all recruits as part of their induction into the military. With the permission of participants, we extracted AFQT percentile scores from military records. AFQT scores are calculated using four subtests (arithmetic reasoning, mathematics knowledge, word knowledge, and paragraph comprehension) from the Armed Services Vocational Aptitude Battery (Xia & Peng, 2024). Latent indicators also included data from four select subtests from the Penn Computerized Neurobehavioral Test Battery (CNB; Moore et al., 2015) administered at T0: go/no-go (Penn Continuous Performance Test, PCPT), list learning (Penn Word Memory Test, PWMT), analogies (Penn Verbal Reasoning Test, PVRT), and face emotion intensity differentiation (Penn Emotion Differentiation Test, PEDT). From these respective tasks, we generated summary measures of attention/processing speed (d-prime), verbal episodic memory (d-prime), language/reasoning (total accuracy), and social cognition (total accuracy).

#### Lifetime prior adversity

At baseline, we also administered the Adverse Childhood Experiences Questionnaire (ACEs; Felitti et al., 1998) and the DRRI-2 Prior Stressors scale (Vogt et al., 2013) to measure cumulative exposure to life stressors prior to BCT. These measures captured a range of adverse experiences, including traumatic events, recalled during childhood and adulthood. Higher scores represented greater stressor exposure. For the present study, internal consistency was acceptable-to-good (ACEs Cronbach’s *α* = .83, DRRI-2 Prior Stressors Cronbach’s *α* = .76). We estimated lifetime prior adversity by combining the binary items from both measures together using two-parameter item response theory (see Supplemental Materials for modeling details).

### Analysis plan

Main study analyses were performed using SPSS Statistics 29. To characterize the study sample, we generated Pearson correlations, means, and standard deviations of the study variables. Given the availability of multiple indicators for our study constructs, we conducted exploratory structural equation modeling, confirmatory factor analysis, and item response theory modeling approaches to synthesize and reduce the dimensionality of our data (R [lavaan 0.6-19], Mplus 8). A more detailed presentation of these procedures is provided in the Supplemental Materials (see Tables S4–S6 and Figure S1).

We conducted a series of hierarchical regression analyses to model resilience as a dynamic, longitudinal process consistent with a developmental psychopathology perspective (https://osf.io/csf4r). Separate models were estimated for each individual difference risk or resilience variable (listwise deletion for missing values). The exception to this were the MPQ scales, which are designed to be used together to maximize measurement differentiation of the personality traits (Patrick et al., 2002; Tellegen & Waller, 2008). As such, PEM, NEM, CON, and absorption were entered simultaneously. As follow-up, we computed hierarchical regression models with each MPQ scale entered individually (see Supplemental Materials, Tables S7–S10). However, no significant risk or resilience effects were observed. In addition, the results of one comprehensive, omnibus model are reported in Supplemental Materials as an additional follow-up to the main analyses (see Table S11).

For the main analyses, in step 1 of each hierarchical model, T1 internalizing distress was regressed onto T0 internalizing distress, perceived BCT stress (BTSS total score), and the individual difference variable(s) of interest. This allowed us to evaluate the main effect of perceived BCT stress on T1 internalizing distress symptoms after adjusting for baseline levels of internalizing symptoms (i.e., to index longitudinal change) and each individual difference variable. In step 2, we tested moderating effects by including an interaction term for BTSS total score -by- each individual difference variable. Predictors were evaluated for significance using non-parametric percentile bootstrap methodology (Wood, 2005). Significance was evaluated at *α* = .008 = .05/6 to account for multiple comparisons (i.e., 99.167% bootstrap resampled confidence interval after 2000 resamples). If significant moderation effects were observed, we had planned to probe them with pick-a-point and Johnson–Neyman approaches.

## Results

Sociodemographic information for the study sample is provided in Table 1. The majority of the sample identified as male with an average age of 19. Approximately two-thirds of the sample identified as White and about 90% as non-Hispanic. The majority of recruits had at least a high school diploma or the equivalent, and the majority were single/not romantically partnered. Table 2 displays correlations among the risk and resilience factors measured at T0, BCT stress, and internalizing distress measured at T0 and T1. At the group level, there was no significant difference in latent internalizing distress from pre-BCT (*M* = .035, *SD* = .829) to post-BCT (*M* = .061, *SD* = .855), *t*(657) = 0.82, *p* = .413, Cohen’s *d* = .03, 95% CI [−.04, .11].

**Table 1.** Baseline sociodemographic and military characteristics of participants

Characteristic	Total post-BCT eligible sample (*n* = 1100)	Study analysis sample (*n* = 657)
Gender, *n* (%)		
Men	768 (69.8)	464 (70.6)
Women	285 (25.9)	181 (27.5)
Age at baseline, *M* (*SD*)	19.0 (3.0)	18.9 (3.0)
Race, *n* (%)		
White	756 (68.7)	457 (69.6)
Black/African American	127 (11.5)	67 (10.2)
American Indian/Alaska Native	47 (4.3)	25 (3.8)
Asian	145 (13.2)	106 (16.1)
Native Hawaiian/Other Pacific Islander	13 (1.2)	8 (1.2)
Other	71 (6.5)	41 (6.2)
Ethnicity, *n* (%)		
Hispanic	114 (10.4)	71 (10.8)
Non-Hispanic	941 (85.5)	575 (87.5)
Highest education level, *n* (%)		
Some high school	382 (34.7)	224 (34.1)
High school diploma/GED	403 (36.6)	252 (38.4)
At least some college	226 (20.5)	139 (21.2)
4-year college+	39 (3.5)	28 (4.2)
Employed outside National Guard, *n* (%)	784 (71.3)	479 (72.9)
Enrolled in school/college, *n* (%)	624 (56.7)	385 (58.6)
Relationship status, *n* (%)		
Single, not partnered	628 (57.1)	386 (58.8)
In relationship, no co-habitation	335 (30.5)	206 (31.4)
In relationship, co-habitation, not married	64 (5.8)	38 (5.8)
Married	30 (2.7)	17 (2.6)
Armed Forces Qualification Test %^ile^, *M* (*SD*)	65.3 (19.8)	66.9 (20.1)

*Note*. *M* = mean, *SD* = standard deviation, GED = General Educational Development test. Sociodemographic characteristics were self-reported at T0 (pre-BCT baseline). Missing self-reports of race are augmented with data from military records. Armed Forces Qualification Test data was extracted from administrative records. Gender was queried as “male,” “female,” “prefer not to answer,” and “don’t know;” only male and female responses were included in these analyses.

**Table 2.** Correlations between variables included in regression analyses

Variable	Internalizing (T0)	Internalizing (T1)	BTSS	MPQ absorption	MPQ PEM	MPQ NEM	MPQ constraint	DERS-16	General cognitive ability	Lifetime Prior Adversity	Social Support
Internalizing (T0)	–	< .001	< .001	< .001	< .001	< .001	< .001	< .001	.911	< .001	< .001
Internalizing (T1)	.516	–	< .001	.001	< .001	< .001	.002	< .001	< .001	< .001	< .001
BTSS	.248	.374	–	.028	.172	< .001	.858	< .001	.001	< .001	.273
MPQ absorption	.210	.129	.081	–	< .001	< .001	.063	< .001	.178	< .001	.552
MPQ PEM	−.353	−.222	−.051	.135	–	.004	.504	< .001	.443	.002	< .001
MPQ NEM	.499	.336	.208	.309	−.091	–	< .001	< .001	< .001	< .001	< .001
MPQ CON	−.126	−.119	.007	−.058	.021	−.205	–	< .001	.019	< .001	< .001
DERS-16	.614	.432	.233	.183	−.348	.472	−.150	–	.309	< .001	< .001
General cognitive ability	.004	.137	.125	−.042	−.024	−.185	−.073	−.032	–	.632	.152
Lifetime prior adversity	.429	.344	.187	.249	−.096	.425	−.173	.323	.015	–	< .001
Social support	−.300	−.206	−.041	.019	.373	−.274	.133	−.211	.045	−.170	–

*Note*. Pearson *r*’s are reported below the diagonal, and *p*s are reported above. T0 = Time 0 (pre-BCT baseline), T1 = Time 1 (post-BCT follow-up), BTSS = Basic Training Stressor Scale, MPQ = Multidimensional Personality Questionnaire, PEM = Positive Emotionality, NEM = Negative Emotionality, CON = Constraint, DERS = Difficulties in Emotion Regulation Scale. Internalizing (T0 and T1), General Cognitive Ability, and Social Support scores were derived from factor analyses; Lifetime Prior Adversity scores were derived from IRT models (see Supplemental Materials). Associations between study variables and gender are depicted in Table S3.

### Do BCT stressor endorsements explain a relative pre- to post-BCT worsening of internalizing symptoms from T0 to T1?

Results from hierarchical regression analyses are reported in Tables 3–8. Across all regression models, we observed several consistent main effects within Step 1. Higher levels of internalizing distress at T0 (pre-BCT baseline) predicted internalizing distress approximately one year later at T1 (post-BCT follow-up; *βs* = .37 − .44). BTSS total scores also predicted greater internalizing distress at post-BCT (*βs* = .25 − .26). Therefore, greater reporting of BCT stressors explained a longitudinal worsening of internalizing distress symptomatology, which could not be explained based solely on recruits’ baseline symptomatology scores.

**Table 3.** Longitudinal prediction of internalizing distress using BTSS and DERS-16

Predictor	*b*	*β*	Bootstrap results		
				99.167% CI_boot_	
			SE	LL	UL
Step 1					
Internalizing distress (T0)	**.381**	**.366**	**.044**	**.259**	**.495**
BTSS-total	**.014**	**.253**	**.002**	**.009**	**.019**
DERS	**.010**	**.133**	**.003**	**.002**	**.018**
Step 2					
BTSS-total × DERS	.00008	.081	.00016	−.00024	.00037

*Note*. T0 = Time 0 (pre-BCT baseline); BTSS = Basic Training Stressors Scale; DERS = Difficulties in Emotion Regulation Scale; *b* = unstandardized predictor coefficient; CI = confidence interval; LL = lower limit; UL = upper limit. Bold values show results that are statistically significant, using a stricter cutoff (*α* = .008) to account for multiple tests. This threshold was determined by dividing the conventional α = .05 by 6 to adjust for multiple comparisons.

**Table 4.** Longitudinal prediction of internalizing distress using BTSS and lifetime prior adversity

Predictor	*b*	*β*	Bootstrap results		
				99.167% CI_boot_	
			SE	LL	UL
Step 1					
Internalizing distress (T0)	**.399**	**.386**	**.040**	**.298**	**.506**
BTSS-total	**.014**	**.254**	**.002**	**.009**	**.019**
Lifetime prior adversity	**.128**	**.137**	**.035**	**.038**	**.220**
Step 2					
BTSS-total × Lifetime prior adversity	−.001	−.028	.002	−.006	.005

*Note*. T0 = Time 0 (pre-BCT baseline); BTSS = Basic Training Stressors Scale; *b* = unstandardized predictor coefficient; CI = confidence interval; LL = lower limit; UL = upper limit. Bold values indicate significant associations with model effects evaluated at *α* = .008 = .05/6 to account for multiple comparisons.

**Table 5.** Longitudinal prediction of internalizing distress using BTSS and general cognitive ability

Predictor	*b*	*β*	Bootstrap results		
				99.167% CI_boot_	
			SE	LL	UL
Step 1					
Internalizing distress (T0)	**.459**	**.444**	**.037**	**.359**	**.554**
BTSS-total	**.013**	**.253**	**.002**	**.008**	**.018**
General cognitive ability	**.090**	**.089**	**.033**	**.000**	**.171**
Step 2					
BTSS-total × general cognitive ability	.002	.093	.002	−.003	.008

*Note*. T0 = Time 0 (pre-BCT baseline); BTSS = Basic Training Stressors Scale; *b* = unstandardized predictor coefficient; CI = confidence interval; LL = lower limit; UL = upper limit. Bold values indicate significant associations with model effects evaluated at *α* = .008 = .05/6 to account for multiple comparisons.

**Table 6.** Longitudinal prediction of internalizing distress using BTSS and MPQ-155

Predictor	*b*	*β*	Bootstrap results		
				99.167% CI_boot_	
			SE	LL	UL
Step 1					
Internalizing distress (T0)	**.387**	**.374**	**.045**	**.260**	**.495**
BTSS-total	**.014**	**.259**	**.002**	**.009**	**.019**
MPQ-PEM	−.004	−.074	.002	−.010	.001
MPQ-NEM	.004	.076	.002	−.002	.009
MPQ-CON	−.004	−.057	.002	−.009	.002
MPQ-absorption	.002	.005	.011	−.027	.031
Step 2					
BTSS-total × MPQ-PEM	.0002	.272	.0001	−.0001	.0004
BTSS-total × MPQ-NEM	.00002	.028	.00011	−.00020	.00021
BTSS-total × MPQ-CON	.0001	.178	.0001	−.0002	.0004
BTSS-total × MPQ-absorption	−.00003	−.006	.00070	−.00145	.00142

*Note*. T0 = Time 0 (pre-BCT baseline); BTSS = Basic Training Stressors Scale; MPQ = Multidimensional Personality Questionnaire; *b* = unstandardized predictor coefficient; CI = confidence interval; LL = lower limit; UL = upper limit; PEM = Positive Emotionality; NEM = Negative Emotionality; CON = Constraint. Bold values indicate significant associations with model effects evaluated at *α* = .008 = .05/6 to account for multiple comparisons.

**Table 7.** Longitudinal prediction of internalizing distress using BTSS and social support

Predictor	*b*	*β*	Bootstrap results		
				99.167% CI_boot_	
			SE	LL	UL
Step 1					
Internalizing distress (T0)	**.441**	**.426**	**.040**	**.338**	**.550**
BTSS-total	**.014**	**.263**	**.002**	**.009**	**.019**
Social support	−.061	−.064	.035	−.154	.038
Step 2					
BTSS-total × social support	.001	.048	.002	−.005	.006

*Note*. T0 = Time 0 (pre-BCT baseline); BTSS = Basic Training Stressors Scale; *b* = unstandardized predictor coefficient; CI = confidence interval; LL = lower limit; UL = upper limit. Bold values indicate significant associations with model effects evaluated at *α* = .008 = .05/6 to account for multiple comparisons.

**Table 8.** Longitudinal prediction of internalizing distress using BTSS and gender

Predictor	*b*	*β*	Bootstrap results		
				99.167% CI_boot_	
			SE	LL	UL
Step 1					
Internalizing distress (T0)	**.434**	**.420**	**.039**	**.329**	**.528**
BTSS-total	**.014**	**.261**	**.002**	**.009**	**.019**
Gender	.146	.077	.067	−.030	.316
Step 2					
BTSS-total × gender	.004	.114	.004	−.007	.015

*Note*. T0 = Time 0 (pre-BCT baseline); BTSS = Basic Training Stressors Scale; *b* = unstandardized predictor coefficient; CI = confidence interval; LL = lower limit; UL = upper limit. Bold values indicate significant associations with model effects evaluated at *α* = .008 = .05/6 to account for multiple comparisons.

### Do pre-BCT individual differences at T0 predict promotive or risk effects on post-BCT internalizing symptoms at T1?

Also within Step 1, we tested potential promotive or risk effects for explaining longitudinal changes above-and-beyond the effects of BCT stressors (i.e., stressor-independent effects). DERS-16 total scores assessed at T0 predicted a worsening of internalizing distress (*β* = .133; Table 3). As such, greater difficulties with self-regulation of emotion was a risk factor for prospective increases in symptoms irrespective of the BCT stressors endorsed by each recruit. Similarly, total lifetime prior adversity loading, as estimated using IRT, predicted a worsening of internalizing distress (*β* = .137; Table 4). Therefore, recall of more lifetime adversity experienced before BCT was also a risk factor for prospective increases in symptoms irrespective of BCT stressors.

Unexpectedly, our latent measure of cognitive ability was also a risk factor (*β* = .089; Table 5), such that greater T0 general cognitive ability predicted a relative worsening in internalizing distress over the course of BCT. No additional individual difference predictors at Step 1 explained promotive- or risk-relevant longitudinal changes in internalizing distress after correction for multiple comparison. Therefore, hypothesized main effects were not supported for the broad traits of personality assessed with the MPQ, social support, and gender (Tables 6–8).

### Do pre-BCT individual differences at T0 predict protective or vulnerability effects on post-BCT internalizing distress at T1?

In Step 2 of the regressions (Tables 3–8), we tested hypothesized protective and vulnerability effects consistent with a modification of the influence of BCT stressors on functioning outcomes. However, no individual difference -by- BTSS total score moderation effects were observed.

## Discussion

This study examined longitudinal changes in internalizing distress psychopathology among US military recruits undergoing BCT, a period of significant life transition and situational challenges. By leveraging this regimented training program, we investigated how individual differences in the frequency and intensity of BCT experiences shaped internalizing symptom outcomes. As expected, self-reported exposure to BCT stressors was associated with worsening internalizing psychopathology over time. Furthermore, we identified three prospective risk factors at baseline for worsening mental health functioning. Two of these risk factors were consistent with hypotheses (greater difficulties in emotion self-regulation, higher lifetime loading of past adversity) while the other (general cognitive ability) was opposite of what we expected. In addition, none of our baseline predictors buffered against or exacerbated the negative consequences of BCT (i.e., no moderation). Taken together, these findings underscore the mental health consequences of BCT-related challenges and how preexisting risk factors may further contribute to the emergence of psychopathology independently of stressors among emerging adults in the military.

The present observational study took advantage of the unique features of BCT as a real-world stressor, which was systematically administered and ecologically valid by design (Polusny et al., 2023). BCT is intended to simulate the demands of military service, functioning both as a training protocol and a structured introduction to military stressors. Although BCT is standardized, recruits can report substantial variability in their experiences (Williams et al., 2016). This variability, captured by BTSS total scores, added essential nuance to our understanding of the mental health outcomes in this sample. Critically, mean-level group changes in internalizing symptoms were not significant from pre- to post-BCT. In other words, the negative psychological impact of BCT would have been hidden had we relied on simpler characterizations of pre–post symptom change alone. Explicitly measuring adversity rather than assuming its uniformity may be important for observing BCT main effects. In addition, the magnitude of BTSS score associations were comparable to those observed in other longitudinal studies of naturalistic stressors and internalizing psychopathology, including during the COVID-19 pandemic (Marquardt et al., 2023), adolescence (Waaktaar et al., 2004), and adulthood (Billings & Moos, 1982). Consequently, BCT appears to be a viable platform for studying the effects of recent life stressors and internalizing symptoms, but with the added scientific benefit of an accelerated timetable for observing the effects (versus waiting for uncontrolled life stressors to happen in everyday life).

Consistent with prior research (Eaton et al., 2011), internalizing psychopathology exhibited some evidence for longitudinal stability. Pre-BCT emotional distress was the strongest study predictor of post-BCT emotional distress – even relative to other variables like BCT stressors. This reinforces the predictive validity of mental health screenings early in the military careers of new recruits (although, see Rona et al., 2005 for commentary). Methodologically, our findings also accentuate the need for pre-stressor baseline measurements to study resilience. An important theme from the longitudinal resilience literature is how post-stressor psychological dysfunction for a significant minority of cases can be better explained by preexisting difficulties (Bonanno et al., 2015; Polusny et al., 2017). When using cross-sectional designs, these more chronically symptomatic individuals become indistinguishable from new onset cases. By including pre-BCT as a model covariate, we statistically isolated variance specific to longitudinal changes as opposed to chronic dysfunction present before BCT stressors occurred.

In addition, main effects of lifetime prior adversity and emotion dysregulation were observed. These findings were consistent with prior research about how early life adversity and self-regulation difficulties are negative predictors of mental health across various populations, including young adults and military populations (Stanley & Larsen, 2019). There are two possible explanations for our prospective findings: (1) Emerging adults with adverse childhood experiences and poor emotion regulation were already on a negative developmental trajectory independent of BCT stressor exposures. Thus, their functioning would have worsened regardless of their military experiences. In contrast, (2) the uniformly delivered aspects of BCT may have caused vulnerability effects (i.e., moderation) to appear like risk (i.e., main) effects. BCT experiences common to all recruits may not be reflected as variability on an instrument like the BTSS but could still have interacted with adverse childhood experiences and emotion regulation difficulties to exacerbate outcomes. An experimental randomization (e.g., BCT vs. waitlist) -by- individual difference (e.g., self-regulatory abilities) statistical design would be needed to further clarify these relationships.

Nevertheless, findings may point towards future pathways for enhancing resilience. Though prior adversity cannot be retrospectively changed, self-regulatory abilities are likely modifiable through intervention (Adler & Gutierrez, 2023). Mindfulness interventions may be particularly applicable for bolstering resilience capacity given how increases in emotion regulation are a known outcome of mindfulness practice (Roemer et al., 2015). Therefore, mindfulness training may function as a preventative intervention if implemented prior to or in the early phases of BCT. However, studies implementing mindfulness among military service members are relatively few in number and lacking in rigorous methodology (Marchand et al., 2021); therefore, additional research is necessary.

Unexpectedly, baseline general cognitive ability was a risk factor as opposed to a promotive or protective factor. This finding is at odds with prior research demonstrating the longitudinal advantages of cognitive ability across the lifespan (M. I. Brown et al., 2021) and how impairments in cognitive ability lead to vulnerability for psychopathology like posttraumatic stress (Thompson & Gottesman, 2008). Military recruits completing BCT may be a unique population in a unique context relative to most prior resilience studies and, therefore, findings may be explained by the developmental psychopathology principles of differential susceptibility and sensitivity to context (Yates et al., 2015). The advantages of general cognitive ability may be dependent on the type of environment people are navigating. With BCT, recruits with greater cognitive ability may struggle with the low level of autonomy and high level of discipline imposed upon them (e.g., low achievement-related subjective control; Pekrun et al., 2010). Therefore, they may perceive BCT as intellectually under-stimulating beyond just being physically and socially taxing. Differential susceptibility also suggests that cognitive ability may not be a static risk factor for internalizing psychopathology at all points in one’s military career. It is possible recruits with higher cognitive ability may experience improved mental health after entering more technical military occupational specialties that match their ability levels.

Contrary to expectations and prior research, we did not observe evidence for several commonly reported resilience-relevant effects. First, despite the hypotheses of stress sensitization and steeling effects – which would predict prior lifetime adversity heightening vulnerability to or protecting against subsequent stressors, respectively (Rutter, 2012) – we found no interaction between lifetime prior adversity and BTSS total scores. Second, we did not observe a buffering effect for social support, which is a well-established resilience factor in longitudinal research (Masten et al., 2021). Social support neither predicted outcomes independently nor moderated the effects of BCT stressors on mental health. Third, although negative emotionality has emerged as a risk and vulnerability factor in past studies (e.g., Masten et al., 1999), MPQ NEM did not significantly moderate the relationship between BCT stressors and internalizing symptoms in our analyses. These various null findings invite consideration of alternative explanations.

One possible contributor to null findings was the co-occurring COVID-19 pandemic, which may have overshadowed BCT as the most salient stressor in recruits’ lives (Salanti et al., 2022). Recruits may have been more distressed by personal stressors (e.g., family, health, financial concerns) than by BCT itself. In addition, not all stressors may interact with resilience resources in the same way. It is possible that only stressors reaching a certain severity or personal relevance level may elicit moderation effects (Geoffrion et al., 2022). Another consideration is the timing and salience of resilience resources. In our study, social support was measured at baseline, but its impact on mental health may depend on its perceived availability during or after the stressors. Although we avoided post-BCT social support measures to preserve temporal precedence, such data might better capture the shifting availability of resilience resources when they matter most. Future work may benefit from modeling how resilience capacity measures may fluctuate across time and stressor contexts.

### Strengths, limitations, and future directions

The current pre-registered study has notable strengths. Our use of BCT as a stressor added to the broader field of resilience science by assessing a real-world yet systematically administered challenge, thereby balancing internal and external validity considerations. In addition, we built upon the methodological limitations of most existing military stress studies (van der Meulen et al., 2020) by using a longitudinal design (versus cross-sectional) and assessing resilience dynamically over time (versus self-reported trait). We also measured cognitive ability using multiple performance-based measures as opposed to self-report of cognitive complaints, which may reduce measurement conflation with current psychological distress (Groenman et al., 2022). Moreover, we measured hypothesized resilience resources at baseline prior to the stressors, which established temporal precedence even if we could not more definitively determine causality with experimental manipulation and random assignment.

Study limitations include the primary reliance on self-report data, which can inflate relationships through mono-method effects. This includes the self-report data about recall of adverse events (e.g., lifetime adversity, perceptions of BCT after the conclusion of BCT) because retroactive reporting about adverse events can be conflated with current internalizing distress (Martin-Wagar et al., 2024; Nelson et al., 2015). Future research may benefit from external measures of military stressors that do not rely on participant memory (Hardt & Rutter, 2004). This would also clarify to what extent this relatively novel BTSS measure differentially reflects objective past events versus biased recall colored by current distress. Moreover, the degree to which recruits were trusting of the assurances of anonymity with National Guard leadership is unknown, which may have altered willingness to share personal details. Finally, results of this study are drawn from one state’s National Guard recruiting battalion and may not generalize to more diverse samples or other military populations.

Considerations for future research include use of a comparison control condition without BCT (e.g., waitlist condition). Control condition(s) would have allowed us to better account for the influence of the COVID pandemic as a simultaneously occurring stressor. Future researchers should also attempt to recruit samples with more profound disruptions in resilience resources to increase the variability available for analyses of these variables (e.g., greater representation of recruits describing poor social support or pathological negative emotionality). Lastly, longer-term follow-up data collection could be used to test if pre-BCT resilience resources have predictive utility for explaining outcomes following deployments and eventual exits from the military. Longer-term follow-up would also allow for an examination of context-dependency of risk and resilience (e.g., when cognitive ability might switch to becoming adaptive in military roles). Our use of one outcome time point limits our ability to test for the variable unfolding for various resilience-related effects.

## Conclusion

This study evaluated potential stressor-independent and stressor-dependent factors among United States Army National Guard recruits undergoing BCT. Using statistical models developed in developmental psychopathology to test resilience as a dynamic process, we found that individual differences in prior exposure to adversity, self-regulatory abilities, and general cognitive ability may influence the experience of internalizing distress in young adults over the course of military training. These findings may pave the way for future research to prospectively enhance outcomes in stressful environments (Luthar & Cicchetti, 2000). This may include clinical trials of early interventions (e.g., mindfulness) to potentially strengthen the psychological functioning of military recruits navigating through significant developmental periods of stress.

## Supporting information

10.1017/S0954579426101199.sm001 McDonald et al. supplementary material McDonald et al. supplementary material

## Data Availability

Authors agree to make data, code, and materials supporting the results or analyses presented in their paper available upon reasonable request and in accordance with institutional policy. It is up to the author to determine whether a request is reasonable.

## Supplementary material

The supplementary material for this article can be found at https://doi.org/10.1017/S0954579426101199.

## Pre-registration statement

This study was pre-registered November 24th, 2023 (https://osf.io/csf4r), and the authors have no deviations to report.

## AI

The authors declare that no generative AI or AI-assisted technologies were used in the writing process.
