# Supplementary material for: Resilience as a dynamic process among military recruits exposed to basic combat training stressors
Source: Dev Psychopathol. 2026 Feb 18:1–13. Online ahead of print. doi: 10.1017/S0954579426101199 (PMC12936461; doi:10.1017/S0954579426101199)
Supplement: McDonald et al. supplementary material [file S0954579426101199sup001.docx]

**Resilience as a dynamic process among military recruits exposed to basic combat training stressors**

***Supplemental Material***

**Table S1**

Attrition Analyses

|  | Insufficient Data | |  | Analysis Sample | |  | T-test | | | |
| --- | --- | --- | --- | --- | --- | --- | --- | --- | --- | --- |
| Variable | *M* | *SD* |  | *M* | *SD* |  | *t* | df | *p* | Cohen *d* |
| Internalizing (T0) | 0.023 | 0.881 |  | 0.032 | 0.827 |  | -0.16 | 1034 | .871 | -0.01 |
| MPQ Absorption | 7.9 | 2.7 |  | 7.7 | 2.7 |  | 1.44 | 1083 | .149 | 0.09 |
| MPQ PEM | **72.8** | **14.4** |  | **71.0** | **14.9** |  | **1.97** | **1079** | **.049** | **0.12** |
| MPQ NEM | 46.5 | 19.0 |  | 44.4 | 17.6 |  | 1.88 | 1079 | .060 | 0.12 |
| MPQ CON | 74.3 | 11.9 |  | 75.0 | 12.7 |  | -0.90 | 1079 | .369 | -0.06 |
| DERS-16 | 31.8 | 12.1 |  | 31.9 | 11.7 |  | -0.21 | 1078 | .837 | -0.01 |
| General Cognitive Ability | **-0.130** | **0.827** |  | **0.103** | **0.846** |  | **-4.41** | **1059** | **<.001** | **-0.28** |
| Lifetime Prior Adversity | 0.016 | 0.894 |  | -0.047 | 0.913 |  | 1.08 | 1025 | .282 | 0.07 |
| Social Support | -0.003 | 0.969 |  | 0.024 | 0.908 |  | -0.47 | 1071 | .639 | -0.03 |

*Note*. Significant group differences in bold. T0 = Time 0 (pre-BCT baseline), MPQ = Multidimensional Personality Questionnaire, PEM = Positive Emotionality, NEM = Negative Emotionality, CON = Constraint, DERS = Difficulties with Emotion Regulation Scale, M = mean, SD = standard deviation. Internalizing, General Cognitive Ability, and Social Support scores were derived from factor analyses. Lifetime Prior Adversity scores were derived from IRT models.

**Table S2.**

Attrition Analyses by Gender

|  | Gender | |  |  |
| --- | --- | --- | --- | --- |
| Eligible Participants | Men (%) | Women (%) |  | Total (%) |
| Insufficient Data (%) | 304 (28.8) | 104 (9.9) |  | 408 (38.7) |
| Analysis Sample (%) | 464 (44.0) | 181 (17.2) |  | 645 (61.2) |
|  |  |  |  |  |
| Total (%) | 768 (72.9) | 285 (27.0) |  | 1054 (100.0) |

*Note*. There was no significant association between gender and the availability of study data for analysis, χ2 = (1, *N* = 1053) = .837, *p* = .260, Cramer's *V* = .028.

**Table S3.**

Associations Between Gender and Other Study Variables

|  | Men | |  | Women | |  | T-test | | | |
| --- | --- | --- | --- | --- | --- | --- | --- | --- | --- | --- |
| Variable | *M* | *SD* |  | *M* | *SD* |  | *t* | df | *p* | Cohen *d* |
| Internalizing (T0) | **-0.078** | **0.772** |  | **0.288** | **0.879** |  | **-5.20** | **643** | **<.001** | **-0.46** |
| Internalizing (T1) | **-0.041** | **0.815** |  | **0.309** | **0.878** |  | **-4.80** | **643** | **<.001** | **-0.42** |
| BTSS | **47.5** | **16.0** |  | **50.8** | **16.1** |  | **-2.37** | **643** | **.018** | **-0.21** |
| MPQ Absorption | 7.7 | 2.6 |  | 7.8 | 2.7 |  | -0.43 | 642 | .668 | -0.04 |
| MPQ PEM | **72.0** | **14.8** |  | **68.8** | **15.1** |  | **2.49** | **642** | **.013** | **0.22** |
| MPQ NEM | 44.0 | 17.6 |  | 44.9 | 17.7 |  | -0.60 | 642 | .548 | -0.05 |
| MPQ CON | **74.4** | **12.6** |  | **76.6** | **12.7** |  | **-2.00** | **642** | **.045** | **-0.18** |
| DERS-16 | **30.5** | **10.8** |  | **35.3** | **13.1** |  | **-4.72** | **629** | **<.001** | **-0.42** |
| General Cognitive Ability | 0.114 | 0.860 |  | 0.075 | 0.814 |  | 0.52 | 640 | .606 | 0.05 |
| Lifetime Prior Adversity | **-0.169** | **0.863** |  | **0.241** | **0.977** |  | **-5.23** | **642** | **<.001** | **-0.46** |
| Social Support | -0.002 | 0.923 |  | 0.134 | 0.817 |  | -1.74 | 643 | .082 | -0.15 |

*Note*. Significant group differences in bold. T0 = Time 0 (pre-BCT baseline), T1 = Time 1 (post-BCT follow-up), MPQ = Multidimensional Personality Questionnaire, PEM = Positive Emotionality, NEM = Negative Emotionality, CON = Constraint, DERS = Difficulties with Emotion Regulation Scale. Internalizing (T0 and T1), General Cognitive Ability, and Social Support scores were derived from factor analyses. Lifetime Prior Adversity scores were derived from IRT models.

**Dimensionality Reductions**

We used exploratory structural equation modeling to reduce the dimensionality of our data (R [lavaan 0.6-19]). As discussed in the main manuscript, this study included measures from the Patient-Reported Outcomes Measurement Information System (PROMIS) system (Cella et al., 2010; Pilkonis et al., 2011), Deployment Risk and Resilience Inventory-2 (DRRI-2; Vogt et al., 2013), Armed Forces Qualification Test (AFQT; Plag & Goffman, 1967), and Penn Computerized Neurobehavioral Test Battery (CNB; Moore et al., 2015). For dimensionality reduction, we examined internalizing symptomatology indicators (Depression-4, PROMIS Anxiety-4, PROMIS Anger-5, PC-PTSD-5) at both baseline (T0) and post-basic combat training (BCT; T1). We assessed social support (PROMIS Instrumental Support-4, PROMIS Informational Support-4, PROMIS Emotional Support-4, DRRI-2 Unit Social Support) and general cognitive ability (AFQT percentile score, CNB Penn Continuous Performance, CNB Penn Word Memory, CNB Penn Verbal Reasoning, CNB Penn Emotion Differentiation) at T0. We used the recommendations of Kline (2016) to assess model fit with exact-fit chi-square statistics and a series of approximate fit statistics: root mean square error of approximation (RMSEA), standardized root mean square residual (SRMR), Tucker–Lewis index (TLI), and Comparative Fit Index (CFI). The level of fit was interpreted using guidelines outlined by Hooper et al. (2007).

## ***Confirmatory Factor Analyses***

For each of the separate unidimensional CFAs, standardized factors loadings were within acceptable ranges for all observed indicators and their respective latent factors (≥ .41; see Table S4). For internalizing distress at T0 and T1, the fit indices of the measurement models demonstrated adequate fit. We also assessed unidimensional for social support at T0 and general cognitive ability at T0. The model fits were deemed fair.

***Measurement Invariance***

Next, we evaluated whether covariation structures for the primary outcome (internalizing symptomatology) were similar at both timepoints by assessing measurement invariance (Brown, 2015). The goal of these analyses was to confirm that the relationship among the measures remained constant before and after BCT (see Table S5). There was no significant change in χ^2^ by constraining the model to have equal factor loadings over time (weak measurement variance). In further support of this, 90% confidence intervals for RMSEA with this constrained model included zero, SRMR was .028. Incremental fit indices were the same or improved relative to the configural models, and both BIC and AIC information criteria improved. However, the addition of equal indicator intercepts for strong invariance resulted in a significant decrease in χ^2^ along with an increase in SRMR, decreases in CFI and TLI, and increases in BIC and AIC. As such, we proceeded forward with our modeling of latent longitudinal change because of this evidence of at least weak measurement invariance over time.

**Table S4**

Confirmatory Factor Analyses for Latent Constructs

| Variable | | Stand Est | *SE* | *z* | *p* | *n* (missing) | χ^2^ (*df*) | *p* | CFI | TLI | RMSEA [90% CI] | SRMR |
| --- | --- | --- | --- | --- | --- | --- | --- | --- | --- | --- | --- | --- |
| Internalizing Distress (T0) | |  |  |  |  | 1133 (6) | 3.745 (2) | .154 | 1.000 | .999 | .028 [.000, .071] | .021 |
|  | Depression-4 | .91 | .01 | 82.43 | <.001 |  |  |  |  |  |  |  |
|  | Anxiety-4 | .90 | .01 | 90.46 | <.001 |  |  |  |  |  |  |  |
|  | Anger-5 | .76 | .01 | 54.18 | <.001 |  |  |  |  |  |  |  |
|  | PC-PTSD-5 | .46 | .04 | 13.01 | <.001 |  |  |  |  |  |  |  |
| Internalizing Distress (T1) | |  |  |  |  | 690 (3) | 0.320 (2) | .852 | 1.000 | 1.001 | .000 [.000, .041] | .008 |
|  | Depression-4 | .94 | .01 | 84.75 | <.001 |  |  |  |  |  |  |  |
|  | Anxiety-4 | .91 | .01 | 82.29 | <.001 |  |  |  |  |  |  |  |
|  | Anger-5 | .80 | .02 | 47.92 | <.001 |  |  |  |  |  |  |  |
|  | PC-PTSD-5 | .53 | .04 | 11.90 | <.001 |  |  |  |  |  |  |  |
| Social Support | |  |  |  |  | 1171 (7) | 2.379 (2) | .304 | 1.000 | .999 | .013 [.000, .061] | .007 |
|  | Emotional Support-4 | .85 | .10 | 31.71 | <.001 |  |  |  |  |  |  |  |
|  | Informational Support-4 | .89 | .09 | 33.15 | <.001 |  |  |  |  |  |  |  |
|  | Instrumental Support-4 | .55 | .11 | 19.16 | <.001 |  |  |  |  |  |  |  |
|  | Unit Social Support | .41 | .29 | 12.35 | <.001 |  |  |  |  |  |  |  |
| General Cognitive Ability | |  |  |  |  | 1161 (7) | 88.917 (5) | <.001 | .907 | .813 | .120 [.099, .143] | .047 |
|  | PWMT (d-prime) | .54 | .03 | 14.96 | <.001 |  |  |  |  |  |  |  |
|  | PCPT (d-prime) | .56 | .04 | 15.58 | <.001 |  |  |  |  |  |  |  |
|  | PEDT (total accuracy) | .46 | .16 | 12.22 | <.001 |  |  |  |  |  |  |  |
|  | PVRT (total accuracy) | .64 | .06 | 18.57 | <.001 |  |  |  |  |  |  |  |
|  | AFQT (percentile) | .73 | .67 | 21.80 | <.001 |  |  |  |  |  |  |  |

*Note*. Stand Est = standardized estimate; CFI = Comparative Fit Index; TLI = Tucker-Lewis Index; RMSEA = Root Mean Square Error of Approximation; CI = confidence interval; SRMR = Standardized Root Mean Square Residual; T0 = Time 0 (pre-BCT baseline); T1 = Time 1 (post-BCT follow-up). Confirmatory factor analyses (CFAs) were conducted separately for each latent variable. Pairwise deletion was used to account for missing data for Internalizing Distress (both T0 and T1). Full information maximum likelihood estimation was used to account for missing data for Social Support. Maximum likelihood estimation was used to account for missing data for General Cognitive Ability.

**Table S5**

Measure Invariance Testing

| Test of Model Fit | Configural (Equal Form) | Weak (Equal Factor Loadings) | Strong (Equal Indicator Intercepts) |
| --- | --- | --- | --- |
| χ^2^ | 23.48 | 25.51 | 43.82 |
| *df* | 15 | 18 | 21 |
| χ^2^_difference_ | -- | 2.42 | 23.02 |
| Δ*df* | -- | 3 | 3 |
| *p*-value | -- | .491 | < .001 |
| Absolute Fit |  |  |  |
| RMSEA (90% CI) | .033 (.000 - .058) | .028 (.000 - .052) | .045 (.025 - .064) |
| SRMR | .026 | .028 | .033 |
| Incremental Fit |  |  |  |
| CFI | .996 | .996 | .989 |
| TFI | .990 | .993 | .981 |
| Information Criteria |  |  |  |
| BIC | 25516.0 | 25500.0 | 25503.8 |
| AIC | 25381.5 | 25379.4 | 25396.2 |

*Note*. RMSEA = Root Mean Square Error of Approximation; CI = confidence interval; SRMR = Standardized Root Mean Square Residual; CFI = Comparative Fit Index; TLI = Tucker-Lewis Index; BIC = Bayesian information criterion; AIC = Akaike’s information criterion.

**Item Response Theory Modeling of Lifetime Prior Stressors**

Total loading of lifetime prior stressors was modeled using two-parameter item response theory (IRT) in Mplus 8 with maximum likelihood estimations (e.g., Marquardt et al., 2023; Patrick et al., 2013). We pooled binary items from the two study measures of prior adversity (ACEs, DRRI-2 Prior Stressors; Felitti et al., 1998; Vogt et al., 2013) using all available cases with sufficient responses at T0 (*n* = 1122). Theta estimates of "ability" in lifetime adversity loading were generated by incorporating each item’s difficulty (i.e., amount of adversity exposure needed to endorse a particular item) and discrimination (i.e., item performance in differentiating people high and low in adversity at that particular difficulty level). This produced an ability value that was very similar, but not identical to the raw total score of the combined ACEs and DRRI-2 Prior Stressors item pool (*r* = .95).

Within the IRT model (Table S3, Figure S1), items with difficulties < 1.1 predominately included household challenges from childhood with several items related to historical emotional maltreatment and physical abuse. Items with difficulties > 1.1 and < 2.3 included personal challenges, physical abuse/neglect, and unwanted sexual abuse and experiences. Items with difficulties > 2.3 included events that would quality as Criterion A trauma as well as one item related to difficulties finding employment. ACEs items accounted for approximately 15% more test information than DRRI-2 Prior Stressors items based on a comparison of the information curves. Furthermore, ACEs items were generally rated by participants as less difficult than DRRI-2 Prior Stressors items.

Mean item discrimination was 1.64 (*SD* = .84) with 87% of the items exhibiting item discriminations above .8 (Table S6, Figure S1). Mean standardized 1-factor CFA loading was .61 (*SD* = .19) with 87% of the items exhibiting loadings above .4. The two worst performing items included having someone close them die (most frequently endorsed) and having to perform dangerous military duties (least infrequently endorsed). The low discrimination values meant that these two poor performing items were downweighed in the overall IRT ability estimates.

In summary, these binary items reflected a plausible continuum of lifetime adversity, with the IRT findings generally reflecting what is known about the relative severity these events. It is well accepted that not all prior adversities are equally important as indicators of lifetime stressor loading (Dharma et al., 2024). This has generated controversy about the appropriateness of modeling lifetime stressor loading as a simple sum of the number of endorsements across various different experiences (e.g., Briggs et al., 2021). The IRT results from this study highlight how there was order and structure in the ways recruits responded to the ACEs and DRRI-2 Prior Stressors questionnaires. Endorsement of higher severity items (e.g., physical abuse) also meant that it was likely those same recruits endorsed lower severity items (e.g., emotional maltreatment), but not vice versa. This is in line with past studies suggesting that ACEs item endorsements are not independent from one another (Dong et al., 2004). As a result, we proceeded forward with extracting the IRT theta estimate for each participant, which we used as a risk predictor in the study regression models.

**Table S6**

Two-parameter Item Response Theory Results for Lifetime Prior Adversity

| **Scale** | **Item #** | **Content** | **Adversity Type** | **CFA 1-factor STDXY Loading** | **IRT Difficulty** | **IRT Discrimination** |
| --- | --- | --- | --- | --- | --- | --- |
| DRRI-2 | 1 | Someone close died | Household Challenges | .180 | -2.579 | .333 |
| DRRI-2 | 9 | Someone close, serious illness/injury, mental health | Household Challenges | .438 | -.552 | .884 |
| ACES | 6 | Parents separated, divorced | Household Challenges | .476 | .105 | .981 |
| ACES | 1A | Adult insulting, humiliation | Emotional Maltreatment | .861 | .364 | 3.068 |
| DRRI-2 | 5 | Caregivers, physical punishment | Physical Abuse | .631 | .411 | 1.476 |
| DRRI-2 | 13 | Financial problems | Household Challenges | .508 | .553 | 1.069 |
| ACES | 1B | Afraid, physical hurt from adult | Physical Abuse | .908 | .712 | 3.923 |
| DRRI-2 | 12 | Emotionally mistreated | Emotional Maltreatment | .758 | .730 | 2.111 |
| ACES | 4B | Family, lack of support | Emotional Maltreatment | .744 | .753 | 2.020 |
| ACES | 2 | Adult, physical | Physical Abuse | .874 | .822 | 3.254 |
| DRRI-2 | 4 | Caregivers, physical fighting | Household Challenges | .700 | .829 | 1.776 |
| ACES | 8 | Household, substance use | Household Challenges | .668 | .952 | 1.626 |
| ACES | 9 | Household, suicide attempt or mental illness | Household Challenges | .606 | .954 | 1.382 |
| ACES | 4A | Family, lack of love | Emotional Maltreatment | .767 | 1.010 | 2.166 |
| DRRI-2 | 14 | Personal physical, mental health problems | Personal Challenges | .634 | 1.234 | 1.489 |
| DRRI-2 | 16 | Childhood, physically hurt by others | Physical Abuse | .771 | 1.539 | 2.196 |
| ACES | 5 | Lack of protection, necessities | Physical Neglect | .760 | 1.633 | 2.122 |
| DRRI-2 | 6 | Childhood, unwanted sexual activity | Sexual Abuse | .731 | 1.688 | 1.946 |
| ACES | 3A | Older person, sexual touch | Sexual Abuse | .774 | 1.702 | 2.216 |
| ACES | 10 | Household member, prison | Household Challenges | .607 | 1.857 | 1.384 |
| ACES | 3B | Older person, sex | Sexual Abuse | .724 | 1.988 | 1.906 |
| DRRI-2 | 7 | Adulthood, unwanted sexual activity | Sexual Abuse | .659 | 2.210 | 1.588 |
| DRRI-2 | 2 | Divorce, left by significant other | Personal Challenges | .417 | 2.214 | .833 |
| DRRI-2 | 17 | Adulthood, physically hurt by others | Physical Abuse | .814 | 2.243 | 2.543 |
| DRRI-2 | 18 | Stressful legal problems | Personal Challenges | .572 | 2.269 | 1.266 |
| DRRI-2 | 10 | Witnessed assault, death | Trauma | .492 | 2.326 | 1.026 |
| DRRI-2 | 11 | Lost job, difficulty finding job | Personal Challenges | .454 | 2.643 | .923 |
| DRRI-2 | 8 | Natural disaster | Trauma | .362 | 2.709 | .705 |
| DRRI-2 | 3 | Robbed, home broken into | Trauma | .362 | 2.947 | .704 |
| DRRI-2 | 15 | Dangerous military duties | Military | .184 | 12.637 | .340 |

*Note*. ACES = Adverse Childhood Experiences scale; DRRI-2 = Deployment Risk and Resilience Inventory-2 Prior Stressors scale; CFA = confirmatory factor analysis; STDXY = standardized independent and dependent variables; IRT = item response theory.

**Figure S1**

Two-parameter IRT Results for Lifetime Prior Adversity


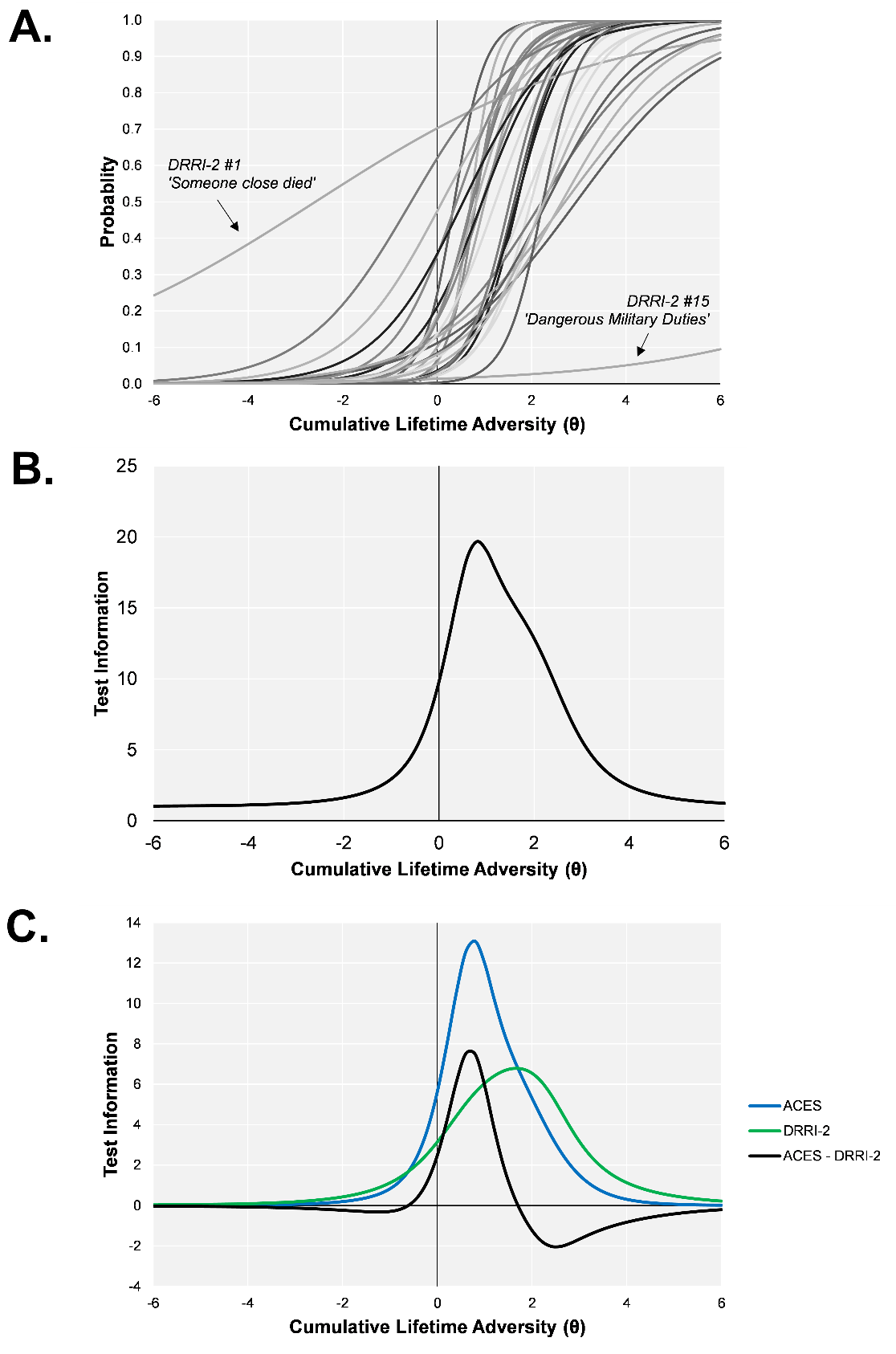


*Note*. Results from the two-parameter item response theory modeling of lifetime prior adversity. (A) Item characteristic curves for the final model combining together items from the Adverse Childhood Experiences (ACES) and Deployment Risk and Resilience Inventory-2 (DRRI-2) Prior Stressors scales. (B) Test information curve for the final model. (C) Test information for the subsets of items from the ACES and DRRI-2 Prior stressors scales with a subtraction metric comparing the two information curves.

**MPQ Scales Entered Individually**

In the main study, we used hierarchical regression to model potential main and moderating effects of personality using the Multidimensional Personality Questionnaire (MPQ; Patrick et al., 2002). In these models, we entered the MPQ scores of interest simultaneously. As follow-up, we also performed four additional models with each MPQ score (Positive Emotionality [PEM], Negative Emotionality [NEM], Constraint [CON], and Absorption) included separately in their own models. Significance was again evaluated at α = .008 = .05/6 to account for multiple comparisons (i.e., 99.167% bootstrap resampled confidence interval after 2000 resamples). However, no significant main or moderation findings consistent with promotive/risk or protective/vulnerability effects were observing using these MPQ variables as separate predictors. See Tables S7-S10.

**Table S7**

Longitudinal Prediction of Internalizing Distress Using BTSS and MPQ-155 PEM

|  |  |  | Bootstrap Results | | |
| --- | --- | --- | --- | --- | --- |
|  |  |  |  | 99.167% CI_boot_ | |
| Predictor | *b* | β | SE | LL | UL |
| Step 1 |  |  |  |  |  |
| Internalizing Distress (T0) | **.439** | **.424** | **.039** | **.326** | **.537** |
| BTSS-total | **.014** | **.264** | **.002** | **.009** | **.019** |
| MPQ-PEM | -.003 | -.060 | .002 | -.009 | .002 |
| Step 2 |  |  |  |  |  |
| BTSS-total x MPQ-PEM | .0002 | .259 | .0001 | -.0001 | .0005 |

*Note.* T0 = Time 0 (pre-BCT baseline); BTSS = Basic Training Stressor Scale; MPQ = Multidimensional Personality Questionnaire; PEM = Positive Emotionality; *b* = unstandardized predictor coefficient; CI = confidence interval; LL = lower limit; UL = upper limit. Bold values indicate significant associations with model effects evaluated at α = .008 = .05/6 to account for multiple comparisons.

**Table S8**

Longitudinal Prediction of Internalizing Distress Using BTSS and MPQ-155 NEM

|  |  |  | Bootstrap Results | | |
| --- | --- | --- | --- | --- | --- |
|  |  |  |  | 99.167% CI_boot_ | |
| Predictor | *b* | β | SE | LL | UL |
| Step 1 |  |  |  |  |  |
| Internalizing Distress (T0) | **.422** | **.408** | **.041** | **.305** | **.531** |
| BTSS-total | **.014** | **.254** | **.002** | **.009** | **.018** |
| MPQ-NEM | .004 | .077 | .002 | -.001 | .009 |
| Step 2 |  |  |  |  |  |
| BTSS-total x MPQ-NEM | .0000 | -.019 | .0001 | -.0003 | .0002 |

*Note.* T0 = Time 0 (pre-BCT baseline); BTSS = Basic Training Stressor Scale; MPQ = Multidimensional Personality Questionnaire; NEM = Negative Emotionality; *b* = unstandardized predictor coefficient; CI = confidence interval; LL = lower limit; UL = upper limit. Bold values indicate significant associations with model effects evaluated at α = .008 = .05/6 to account for multiple comparisons.

**Table S9**

Longitudinal Prediction of Internalizing Distress Using BTSS and MPQ-155 CON

|  |  |  | Bootstrap Results | | |
| --- | --- | --- | --- | --- | --- |
|  |  |  |  | 99.167% CI_boot_ | |
| Predictor | *b* | β | SE | LL | UL |
| Step 1 |  |  |  |  |  |
| Internalizing Distress (T0) | **.451** | **.436** | **.037** | **.347** | **.544** |
| BTSS-total | **.014** | **.263** | **.002** | **.009** | **.019** |
| MPQ-CON | -.004 | -.065 | .002 | -.010 | .001 |
| Step 2 |  |  |  |  |  |
| BTSS-total x MPQ-CON | .0001 | .196 | .0001 | -.0002 | .0005 |

*Note.* T0 = Time 0 (pre-BCT baseline); BTSS = Basic Training Stressor Scale; MPQ = Multidimensional Personality Questionnaire; CON = Constraint; *b* = unstandardized predictor coefficient; CI = confidence interval; LL = lower limit; UL = upper limit. Bold values indicate significant associations with model effects evaluated at α = .008 = .05/6 to account for multiple comparisons.

**Table S10**

Longitudinal Prediction of Internalizing Distress Using BTSS and MPQ-155 Absorption

|  |  |  | Bootstrap Results | | |
| --- | --- | --- | --- | --- | --- |
|  |  |  |  | 99.167% CI_boot_ | |
| Predictor | *b* | β | SE | LL | UL |
| Step 1 |  |  |  |  |  |
| Internalizing Distress (T0) | **.458** | **.443** | **.037** | **.353** | **.551** |
| BTSS-total | **.014** | **.261** | **.002** | **.009** | **.019** |
| MPQ-Absorption | .002 | .006 | .010 | -.023 | .029 |
| Step 2 |  |  |  |  |  |
| BTSS-total x MPQ-Absorption | .0004 | .081 | .0007 | -.0016 | .0022 |

*Note.* T0 = Time 0 (pre-BCT baseline); BTSS = Basic Training Stressor Scale; MPQ = Multidimensional Personality Questionnaire; *b* = unstandardized predictor coefficient; CI = confidence interval; LL = lower limit; UL = upper limit. Bold values indicate significant associations with model effects evaluated at α = .008 = .05/6 to account for multiple comparisons.

**All Study Predictors Entered Simultaneously**

As follow-up, we also executed one comprehensive, omnibus model with the various resilience-relevant variables included simultaneously as predictors (see Table S11). Significance was again evaluated at α = .008 = .05/6 (i.e., 99.167% bootstrap resampled confidence interval) to facilitate direct comparison to the models reported in the main manuscript. T0 internalizing distress continued to be a positive predictor of T1 internalizing distress. Similarly, BTSS total scores also predicted T1 internalizing distress. At Step 1, only general cognitive ability remained a significant predictor of T1 internalizing distress. We observed no moderation effects within Step 2.

Although we had theoretical interest in examining these variables in the main study, we recognized that they were not designed explicitly to work together. It is likely that several of these resilience-relevant predictors capture overlapping variance of interest for predicting internalizing outcomes (see Table 2 bivariate correlations from the main manuscript). This is especially true for variables like DERS-16 and MPQ-NEM, which were all correlated r = .472. In contrast, general cognitive ability has much smaller associations with the other predictors and may not have been as affected through the covariation procedures in this larger regression model.

**Table S11**

Omnibus Model

|  |  |  | Bootstrap Results | | |
| --- | --- | --- | --- | --- | --- |
|  |  |  |  | 99.167% CI_boot_ | |
| Predictor | *b* | β | SE | LL | UL |
| Step 1 |  |  |  |  |  |
| Internalizing Distress (T0) | **.279** | **.268** | **.049** | **.142** | **.403** |
| BTSS-total | **.012** | **.235** | **.002** | **.008** | **.018** |
| Gender | .145 | .076 | .067 | -.031 | .334 |
| Lifetime Prior Adversity | .087 | .094 | .036 | -.007 | .184 |
| General Cognitive Ability | **.100** | **.099** | **.033** | **.011** | **.191** |
| DERS-16 | .007 | .090 | .003 | -.002 | .015 |
| Social Support | -.055 | -.058 | .038 | -.163 | .046 |
| MPQ-PEM | -.002 | -.041 | .002 | -.008 | .004 |
| MPQ-NEM | .004 | .072 | .002 | -.003 | .010 |
| MPQ-CON | -.001 | -.019 | .002 | -.008 | .005 |
| MPQ-Absorption | -.002 | -.008 | .011 | -.031 | .025 |
| Step 2 |  |  |  |  |  |
| BTSS-total x Gender | .004 | .128 | .004 | -.007 | .015 |
| BTSS-total x Lifetime Prior Adversity | -.001 | -.040 | .003 | -.008 | .005 |
| BTSS-total x General Cognitive Ability | .002 | .088 | .002 | -.004 | .008 |
| BTSS-total x DERS-16 | .0002 | .189 | .0002 | -.0003 | .0007 |
| BTSS-total x Social Support | -.002 | -.105 | .002 | -.008 | .004 |
| BTSS-total x MPQ-PEM | .0003 | .531 | .0001 | -.0001 | .0007 |
| BTSS-total x MPQ-NEM | .0000 | .026 | .0001 | -.0004 | .0004 |
| BTSS-total x MPQ-CON | .0001 | .215 | .0002 | -.0003 | .0006 |
| BTSS-total x MPQ-Absorption | -.0003 | -.074 | .0007 | -.0022 | .0015 |

*Note.* T0 = Time 0 (pre-BCT baseline); BTSS = Basic Training Stressor Scale; MPQ = Multidimensional Personality Questionnaire; *b* = unstandardized predictor coefficient; CI = confidence interval; LL = lower limit; UL = upper limit. Bold values indicate significant associations with model effects evaluated at α = .008 = .05/6.
